# Digital health literacy framework for PE teachers: a grounded theory study

**DOI:** 10.3389/fpubh.2026.1771020

**Published:** 2026-04-28

**Authors:** Yu Yang, Weijin Shi

**Affiliations:** School of Physical Education, Shanghai Normal University, Shanghai, China

**Keywords:** data ethics in education, digital health literacy, digital teaching competence, grounded theory, physicaleducation teachers

## Abstract

**Background:**

As digital technologies transform health information environments, digital health literacy has become a core professional competence for school-based health education. Physical education (PE) teachers serve as the primary facilitators of school health promotion; however, unlike general educators, they are uniquely responsible for bridging physical activity with complex digital health data, making their specific competency requirements critical yet undertheorised.

**Methodology:**

This qualitative study used a grounded theory approach to conduct semistructured interviews with 34 participants (primary/secondary school PE teachers and higher education experts) from 12 provinces in China. Interview data were analyzed with NVivo 15.0 through open, axial and selective coding, which was supplemented by relevant literature and policy documents.

**Results:**

The analysis generated a Digital Health Literacy Framework for PE Teachers. It comprises three core dimensions: Digital Health Awareness and Critical Literacy, Digital Teaching Practice and Innovative Application Competence and Digital Security Ethics and Environmental Awareness Competence. Distinct from general digital literacy models, this framework specifically integrates pedagogical content knowledge with health-related information appraisal, capturing the intersection of technology, health promotion, and physical education pedagogy.

**Conclusion:**

The proposed framework conceptualizes PE teachers' digital health literacy as a systematic competency system. It provides an evidence-based foundation for developing national standards, training modules, and digitalization policies.

## 1 Introduction

Within the contemporary convergence of global public health and education policies, rapid advances in digital technologies are reshaping the ways health information is produced, disseminated and interpreted (1). This transformation aligns with arguments by digital health literacy scholars that health competence now depends on technological engagement and critical evaluation skills (2–5). While existing literature has extensively mapped the digital health literacy (DHL) of patients and students, there remains a critical research lacuna regarding the “professional digital health literacy” of educators who are expected to facilitate these competencies. The World Health Organisation's (WHO) Global Digital Health Strategy (2020–2025) identifies improved digital health literacy as essential for mitigating health inequalities, signaling a shift toward competency-based approaches (6, 7). Correspondingly, global policy trends increasingly position schools as key sites for cultivating integrated health and digital competencies (8–10). Building on this foundation, this study extends the DHL discourse into the specific professional domain of school-based health education.

Schools, particularly at the primary and secondary levels, are central arenas for advancing this new educational mission. Within this setting, the role of physical education (PE) teachers is undergoing a notable paradigm shift. Specifically, PE teachers serve as the primary facilitators of school health promotion because they are uniquely positioned to bridge physical activity with complex digital health data. As reflected in the Health Promoting Schools framework, teachers are increasingly positioned as holistic facilitators of students' physical, cognitive and digital wellbeing (11). Rather than serving solely as instructors of motor skills, they are becoming ‘bridge-builders' who connect the digital health domain with students' embodied practices, thus guiding the development of lifelong health behaviors. This “embodied” transition is critical, as it transforms abstract online information into physical habit. Therefore, their professional competence extends beyond pedagogical and physiological knowledge to include data interpretation, digital platform navigation and critical appraisal of online health content (12, 13). However, this expanding role raises a fundamental question that remains insufficiently theorized: what is the underlying structure of digital health literacy required of PE teachers in the digital era? Existing frameworks are often subject-neutral, creating a theoretical gap regarding the specific pedagogical-technical intersection of PE. The absence of a clear conceptual model constitutes a significant bottleneck for theoretical development and practical innovation in this emerging field.

Digital health literacy has evolved from a static cognitive skill to a dynamic, context-responsive competence that is shaped by digital environments (14–16). It builds on functional, interactive and critical health literacy, thereby requiring individuals not only to access but also to evaluate critically and apply digital health information across platforms (17, 18). The European Commission similarly stresses technical and critical capacities (19). However, most studies rely on general frameworks or equate digital health literacy with technology use, thus overlooking pedagogical demands in school contexts (20, 21). The novelty of this study lies in addressing how PE teachers convert abstract digital health information into embodied, actionable behaviors—a dimension largely underexplored in existing theoretical models.

### 1.1 Theoretical Narrative and Conceptual Framework

To illustrate the pathways for the research outcomes, we propose a conceptual framework where teacher-level competencies (explanatory variables) drive student-level health outcomes. This framework integrates four interlinked theories: From a social interaction perspective, Collins' (22) interaction ritual chain theory explains how meaningful social engagement generates emotional energy and group cohesion. In digital health education, this concept suggests that PE teachers can transform abstract health knowledge into tangible behavioral commitment by designing ritual-like digital experiences that cultivate belonging and shared purpose (23, 24). Several practices, such as digital health challenges or online health communities, enable students to exchange experiences, offer mutual encouragement and collaboratively address health-related tasks, thereby strengthening engagement and digital health literacy (25–27). Accordingly, PE teachers' digital health literacy necessarily includes the ability to design and facilitate such digital interaction rituals.

Students' cognitive and learning differences require differentiated instruction supported by digital tools. Gardner's multiple intelligences theory helps explain why PE teachers' digital health literacy must include recognizing diverse learner strengths and adapting instruction accordingly (28). Appropriately selected digital platforms can align health content with students' varied learning profiles, thereby enhancing the inclusiveness and effectiveness of digital health education (29, 30). This adaptation serves as a key explanatory variable for personalized health outcomes.

The TPACK framework underscores that effective technology integration requires the coordinated use of technological, content and pedagogical knowledge. When it is applied to PE, it suggests that digital health literacy entails the capacity to align digital tools with health-related content and pedagogical aims, thus enabling informed and contextually appropriate instructional decisions (31). This provides the structural foundation for our framework.

From a motivational perspective, self-determination theory posits that intrinsic motivation is strengthened when the psychological needs of autonomy, competence and relatedness are met. When it is applied to digital health education, it suggests that students are likely to engage sustainably in healthy behaviors when digital tools support personalized competence feedback, offer meaningful choice and foster a sense of connection through digital communities (32, 33). In our model, the teacher's capacity to design these motivationally supportive digital ecosystems acts as the primary driver for the final outcome: sustainable student health behavior.

In summary, the digital health literacy that is required of primary and secondary school PE teachers is a complex, multidimensional construct that is shaped by the demands of the digital era and embedded in embodied teaching practice. It encompasses capacities for information appraisal, the facilitation of digital interaction, the adaptation of teaching strategies and ethical engagement with health data. However, no existing theoretical model has adequately captured this multifaceted structure, which leaves assessments of PE teachers' digital health competencies without a clear conceptual foundation and limits the development of coherent preservice and in-service professional training.

Given the conceptual ambiguity and the emerging nature of this field, this study adopts grounded theory, which was originally developed by Glaser and Strauss in 1967, as its primary methodological approach. As an inductive qualitative method, grounded theory enables theoretical concepts to emerge directly from empirical data rather than from predetermined assumptions (34–37). It provides a structured yet flexible process in which theoretical insights develop progressively through iterative data collection, constant comparison and coding (38). Notably, grounded theory constitutes a methodological pathway rather than a fixed theoretical system. Over time, Strauss's version has become the most widely cited, employing three progressive coding procedures—open, axial and selective coding—to integrate data into a coherent theoretical framework (39, 40). This discovery-oriented process is particularly valuable in fields where conceptual foundations remain underdeveloped (41), supporting the construction of a data-driven framework through iterative data collection and systematic coding.

## 2 Materials and methods

### 2.1 Research objectives

The overall objective of this study is to construct a comprehensive and theoretically grounded framework of digital health literacy specifically tailored for primary and secondary school PE teachers. To achieve this, the study pursues three specific objectives: (1) to identify the core dimensions of digital health literacy within the context of physical education; (2) to operationalise these dimensions through empirical categories and concepts derived from frontline practice; and (3) to provide a systematic theoretical basis for future competency assessment and professional development.

### 2.2 Participants

To ensure methodological rigor, this study employed a “Maximum Variation Sampling” strategy to recruit participants across diverse geographical and professional backgrounds. Consistent with grounded theory principles, theoretical sampling was adopted, which emphasized purposeful selection and relatively small sample sizes to capture diverse and conceptually relevant perspectives. Accordingly, specific measures were taken to ensure diversity across 12 provinces (representing varying economic and educational development levels in China) and school levels (balanced between primary, junior high, and senior high schools).

Participants were selected based on their ability to provide experience-based insights into primary and secondary school PE teachers' digital health literacy (42). Two groups were recruited: (1) frontline PE teachers who were directly engaged in teaching practice and (2) experts and scholars in PE and health education who offered broad theoretical perspectives. The final sample size of 34 participants was determined by the principle of theoretical saturation. While qualitative studies often reach thematic saturation with 12–20 interviews, we extended the sample to 34 to ensure that the unique socio-cultural nuances of different provinces and the specific pedagogical demands of different school levels were fully captured. Grounded theory typically relies on small samples that are aimed at reaching theoretical saturation; the sample size is determined by informational depth rather than numerical adequacy (43). We confirmed saturation when the final three interviews yielded no additional concepts or categories, and the internal relationships within the established framework remained stable. In total, 34 participants from 12 provinces across North China, East China, South China, Southwest China and Northwest China were interviewed. We ensured anonymity by assigning numerical identifiers (P1–P34) to all participants. Table 1 presents the demographic information in detail.

**Table 1:** Basic information of interview participants (*N* = 34).

No.	Form	Title	Location	Instrument
1	Individual interviews	PE teachers in primary and secondary schools	Eastern China	Tencent meeting app
2	Individual interviews	PE teachers in primary and secondary schools	Southern China	Tencent meeting app
3	Individual interviews	PE teachers in primary and secondary schools	Western China	Tencent meeting app
4	Individual interviews	PE teachers in primary and secondary schools	Western China	Tencent meeting app
5	Individual interviews	PE teachers in primary and secondary schools	Northern China	Tencent meeting app
6	Individual interviews	PE teachers in primary and secondary schools	Eastern China	Phone recording app
7	Individual interviews	PE teachers in higher education	Eastern China	Email
8	Individual interviews	PE teachers in primary and secondary schools	Central China	Tencent meeting app
9	Individual interviews	PE teachers in higher education	Eastern China	Email
10	Individual interviews	PE teachers in primary and secondary schools	Eastern China	Tencent meeting app
11	Individual interviews	PE teachers in primary and secondary schools	Northern China	Tencent meeting app
12	Individual interviews	PE teachers in primary and secondary schools	Northern China	Tencent meeting app
13	Individual interviews	PE teachers in primary and secondary schools	Eastern China	Tencent meeting app
14	Individual interviews	PE teachers in primary and secondary schools	Northern China	Tencent meeting app
15	Individual interviews	PE teachers in primary and secondary schools	Eastern China	Voice recorder
16	Individual interviews	PE teachers in primary and secondary schools	Eastern China	Email
17	Individual interviews	PE teachers in higher education	Eastern China	Email
18	Individual interviews	PE teachers in primary and secondary schools	Eastern China	Email
19	Individual interviews	PE teachers in primary and secondary schools	Eastern China	Voice recorder
20	Individual interviews	PE teachers in primary and secondary schools	Eastern China	Voice recorder
21	Individual interviews	PE teachers in primary and secondary schools	Eastern China	Email
22	Individual interviews	PE teachers in primary and secondary schools	Eastern China	Email
23	Focus group	PE teachers in primary and secondary schools		Tencent meeting app
24	Focus group	PE teachers in primary and secondary schools		Tencent meeting app
25	Group interviews (8 persons)	PE teachers in primary and secondary schools		Voice recorder

### 2.3 Interview

An interview guide titled ‘Exploring the Structure of Digital Health Literacy Among Primary and Secondary School Physical Education Teachers' was developed to address the research objectives. The guide was first piloted with three representative PE teachers to ensure methodological rigor. Based on the teachers' feedback and following consultations with two experts in school PE and health education, two rounds of revisions were conducted to refine the content, logical structure and wording.

Given the geographical dispersion of participants across multiple provinces, data were collected through a combination of face-to-face interviews and online video conferencing. Interview appointments were arranged in advance. Then, the interview guide was sent to participants beforehand to facilitate preparation and ensure the depth of responses. In-person interviews were conducted in quiet, interruption-free environments with each session lasting approximately 40–60 min.

Throughout the interview process, standard qualitative research ethics were strictly observed. Rapport was established to create a psychologically safe atmosphere, thus enabling participants to express their experiences and perspectives openly. Semistructured interviews allowed for flexibility: while centered on core questions, the sequence was adapted based on participants' responses. Leading questions were deliberately avoided. Instead, active listening and probing techniques were employed to elicit specific behaviors, contextual details and illustrative examples. For instance, when participants reported using digital health tools in PE teaching, follow-up questions explored tool types, usage scenarios, student reactions and observed teaching outcomes. This approach ensured the collection of rich, detailed data that were necessary for analyzing and theorizing the structure of PE teachers' digital health literacy.

### 2.4 Data analysis

Following each interview, audio recordings were transcribed verbatim to ensure accuracy and preserve the integrity of participants' original statements. In total, 34 transcripts comprising approximately 120,000 words were generated. Qualitative data analysis software NVivo 15.0 was used to organize and code the data systematically.

The integration of policy documents into the coding process was conducted systematically rather than selectively. In addition to interview data, a corpus of 15 national-level policy documents related to school PE were incorporated to enrich the analytical context. These documents were treated as supplementary “textual participants” and subjected to the same three-stage coding procedure (open, axial, and selective coding) as the interview transcripts. Throughout the analysis, codes were iteratively revised through constant comparison between empirical interview data and official policy requirements to refine categories and ensure analytic rigor. This process facilitated the development of a comprehensive framework of digital health literacy among primary and secondary school PE teachers.

#### 2.4.1 Open coding

Open coding, which is the first and foundational stage of the coding process in grounded theory, involves systematically examining the raw data to identify and label meaningful units. This stage requires careful, line-by-line analysis to ensure that potentially relevant information is not overlooked. It comprises three core processes: labeling, conceptualization and categorization.

During labeling, which is also referred to as preliminary conceptualization, interview data were inductively analyzed and compared to generate concise statements that captured the essential meaning of participants' expressions (see Table 2). Each statement reflected a distinct aspect of digital health literacy as articulated by interviewees while preserving the original semantic intent. Through this process, a total of 214 statements that were relevant to primary and secondary school PE teachers' digital health literacy were identified. These statements were recorded using the naming convention “EN + statement number” (e.g., E5 Acquiring digital health information through exchanges with colleagues or peers; E206 Quantifying teaching evaluations through digital health tools) (see Table 3).

**Table 2:** Example of the labeling process for raw data.

Data source	Original data	Labeling
Interview materials numbered 6	I utilize digital health tools in my daily teaching practice to guide students. One specific application involves employing fitness applications to record students‘ exercise data in real time. These applications not only track metrics such as activity levels, heart rate, and calorie expenditure but also 3provide personalized health recommendations. In class, I guide students to understand their exercise status by displaying this data in real time, helping them set appropriate exercise goals. Furthermore, this data enables me to assess students' physical fitness more accurately and develop more suitable training programmes tailored to each student's characteristics.	E35 Assessing digital health information apps to evaluate student exercise data. E49 Utilizing digital health tools to alleviate teaching workload. E51 Assisting students in setting exercise goals via digital health tools. E57 Optimizing exercise training plans through digital health tools. E59 Enhancing the scientific rigor of teaching evaluations through digital health tools. E206 Quantifying teaching evaluations through digital health tools.

**Table 3:** Examples of 214 tagged labels result.

Labels
E1 Acquiring digital health information through academic journals
E2 Obtaining digital health information through online courses
E3 Obtaining digital health information via social media
E4 Obtaining digital health information through government or education authority channels
E5 Acquiring digital health information through exchanges with colleagues or peers
E6 Acquiring digital health information through industry seminars or academic conferences
……
E32 Evaluating the effectiveness of digital health tools through trial use
E33 Testing digital health tools via small-scale pilot programmes
E34 Monitoring students' heart rates using fitness trackers
E35 Assessing digital health information apps to evaluate student exercise data
E36 Employing smart devices to support classroom teaching
E37 Utilizing motion capture or correction technology to assist instruction
……
E205 Assessing improvements in student health through digital health data
E206 Quantifying teaching evaluations through digital health tools
E207 Personalizing teaching evaluations through digital health tools
E208 Comparing individual student progress using digital health tools
E209 Analyzing class-wide physical activity levels via digital health tools
E210 Integrating digital health tools with traditional assessment methods
E211 Applying digital health tools in physical education teaching for secondary school or university entrance examinations
E212 Adapting digital health tools for teaching in volunteer support or frontier regions
E213 Optimizing digital health tool implementation based on student feedback
E214 Collaborating with colleagues to refine operational procedures for digital health tools

Conceptualization involves refining and consolidating the labeled statements according to principles of validity, similarity and relevance. Statements with closely related meanings were grouped to form high-level concepts (see Table 4). Through this process, 40 concepts were distilled from the initial 214 labeled statements. These concepts were recorded using the convention “DN + concept name” (e.g., D4 Capable of extending postclass physical exercises and implementing digital follow-up management) (see Table 5). To further consolidate these findings into a more manageable structure, these concepts were systematically grouped through the final step of open coding: categorization.

**Table 4:** Example of conceptualization process from 214 labels.

Result of conceptualization	Label reference	Original data reference
D17 Feedback on teaching effectiveness and process optimisation with a focus on tool application	E95 Communicate with digital health tool developers	You could enquire with the developers of health apps about the principles behind their testing. For instance, last time there was an app claiming to correct spinal alignment. I rang their customer service directly to ask whether the algorithm team possessed any medical background. They stammered and couldn't provide a clear answer, so I promptly decided against using it.
	E96 Adjusting teaching programmes when digital health tools fail to meet expected outcomes	Should outcomes fall short of expectations, one must first adjust lesson plan design. For instance, if exercise load or intensity proves inadequate, enhancing intensity within the lesson plan is paramount to achieving desired objectives.
	E99 Simplifying usage procedures when digital health tools prove overly complex	As an experienced teacher, my demand for digital health tools is low, primarily because installing and operating the equipment wastes class time. Schools lack fixed, sufficient equipment, requiring queuing for use, which disrupts teaching progress. However, I recognize the tools' advantages and hope schools will provide adequate equipment and simplify operational procedures in future to facilitate classroom use.
		E214 Collaborate with colleagues to refine operational procedures for digital health tools	I would engage in peer discussions and knowledge sharing. As a new teacher, I would learn from experienced colleagues, exchanging insights and perspectives on the practical application of digital health tools.

**Table 5:** Examples of 40 concept results.

Concepts generated from conceptualization process	Label References
D1 Capable of acquiring digital health information from multiple channels	E1, E2, E3, E4, E5, E6, E7, E8, E9, E10, E11, E12, E13, E14, E15
D2 Ability to evaluate and verify information content	E16, E17, E18, E19, E20, E21, E22, E23, E24, E25, E97, E98, E155
D3 Capable of selecting and evaluating educational technology tools	E26, E27, E28, E29, E30, E31, E32, E33, E69, E75
D4 Capable of extending post-class physical exercises and implementing digital follow-up management	E40, E54, E113, E149
……	……
D37 Capable of addressing issues encountered when using digital health tools	E100, E101, E102, E103, E104, E105
D38 Ability to enhance digital health literacy through multiple channels	E156, E157
D39 Possesses clear functional requirements for digital health tools in teaching	E181, E182
D40 Focuses on identifying limiting factors in the application of digital health technologies	E136, E138

Categorization refers to deriving overarching categories from the established concepts, assigning appropriate category labels and ensuring that each category remains conceptually distinct while sufficiently encompassing its constituent concepts (see Table 6). Through this process, eight categories were generated from the 40 concepts. These categories follow the naming convention “CN + category name” (e.g., C8 Digital teaching technology requirements outlook and functional critique literacy)(see Table 7).

**Table 6:** Example of conceptualization process from 40 concepts.

Result of categorization	Conceptualization reference	Labeling reference	Original data reference
C5 Digital Collaboration and Resource Integration Literacy	D10 Ability to Integrate and Apply Interdisciplinary Teaching Resources	E88 Collaborating with cross-disciplinary teachers to deliver digital health instruction	Sport itself is inherently interdisciplinary, encompassing fields such as exercise physiology and psychology. When analyzing sports injury data with the school doctor, we observed a general lack of flexibility in one particular class, prompting us to incorporate stretching exercises into the curriculum.
		E159 Enhancing Digital Health Literacy Through Interdisciplinary Learning	I engage in exchanges with colleagues regarding digital health initiatives, such as sharing insights on fitness tracker usage during teaching and research group sessions, and discussing how to adapt teaching approaches based on data. I also collaborate with teachers from other subjects, for instance, discussing with mathematics teachers how to integrate fitness data into statistical teaching to enhance pupils' comprehensive skills.
	……	……	……
	D32 Emphasis on collaborative expansion models for digital teaching resources	E142 Acquiring Digital Health Resources Through Industry-Academia Collaboration	We hope to enhance privacy protection technologies for digital health solutions and improve data security. We also hope that schools will enter into contracts with relevant parties to safeguard the privacy of students and staff, thereby preventing data misuse.
		E143 Acquiring Digital Health Resources Through Inter-Institutional Collaboration	Organize student case studies in the classroom to explore digital health application scenarios (such as discrepancies in fitness app data); host seminars inviting experts to share technological developments while encouraging staff and students to exchange usage insights; conduct collaborative research with institutions and enterprises (e.g., “Practical Application of AI Motion Correction Systems in Basketball Instruction”) and share findings; attend academic conferences to deliver oral presentations on digital health teaching research and receive peer feedback.

**Table 7:** Examples of 8 categories result.

Categories	Concepts	Number of labels
C1 Digital information processing and evaluation literacy	D1 Ability to access digital health information from multiple channels	15
	D2 Capable of conducting information content evaluation and verification practices	13
	D22 Possesses an intrinsic understanding of digital health literacy	10
	D37 Capable of addressing issues encountered when using digital health tools	6
	D38 Capable of enhancing digital health literacy through multiple avenues	2
……	……	……
C8 Digital teaching technology requirements outlook and functional critique literacy	D27 Prospects for functional requirements of teaching tools	5
	D39 Clear functional requirements for digital health tools in teaching	2

#### 2.3.2 Axial coding

Building on the eight categories derived from open coding, the second stage of the analysis involved axial coding. Axial coding, which is the second and central stage of the coding process in grounded theory, involves reestablishing connections among the categories that were generated during open coding. This stage requires a detailed comparison and analysis of intercategory relationships to identify high-level primary categories. Through axial coding, three primary categories were identified, following the naming convention “BN + category name”: B1 Digital Health Awareness and Critical Literacy, B2 Digital Teaching Practice and Innovative Application Competence and B3 Digital Security Ethics and Environmental Awareness Competence (see Table 8).

**Table 8:** Eight categories derived from axis coding.

Main categories	Sub-categories
B1 Digital Health Awareness and Critical Literacy	C1 Digital Information Processing and Evaluation Literacy
	C6 Critical Reflection and Digital Sustainability Literacy
	C8 Digital Teaching Technology Requirements Outlook and Functional Critique Literacy
B2 Digital Teaching Practice and Innovative Application Competence	C2 Digital Tool Application and Optimisation Competence
	C3 Digital Instruction Monitoring and Data Analysis Competence
	C5 Digital Collaboration and Resource Integration Competence
B3 Digital Security Ethics and Environmental Awareness Competence	C4 Data Security and Ethical Standards Competence
	C7 Digital Instructional Environment and Implementation Feasibility Awareness

#### 2.3.3 Selective coding

Once the hierarchical relationships between categories were clarified through axial coding, the analysis culminated in the selective coding stage. Selective coding represents the final stage of grounded theory analysis during which the “core category” is identified and systematically linked to all other categories. This stage involves validating intercategory relationships, integrating categories whose conceptualization remains incomplete and elevating the analysis to a high level of abstraction. Building on the previously identified categories, continual comparison with the original data enabled the synthesis of all elements into the overarching core category: Digital Health Literacy of Primary and Secondary School Physical Education Teachers.

### 2.4 Theoretical saturation test

A systematic theoretical saturation test was conducted to ensure the rigor and completeness of the theoretical framework (44). The purpose of this test was to determine whether the constructed Structural System of Digital Health Literacy for Physical Education Teachers sufficiently captured all essential categories and properties of the phenomenon. Consistent with qualitative research standards, theoretical saturation was defined as the point at which additional data no longer produced new concepts or category attributes.

For validation, all interview transcripts were randomly divided into two sets: approximately 80% of the data served as the primary analytic corpus, while the remaining 20% were reserved as a saturation test sample. Coding of the validation sample in NVivo 15.0 revealed no new concepts, categories or relational structures. The existing model adequately accounted for all relevant information contained in the reserved data. These procedures confirm that the core theoretical construct developed in this study has reached saturation, thereby demonstrating strong completeness and methodological rigor (45).

## 3 Results

**Figure 1:**
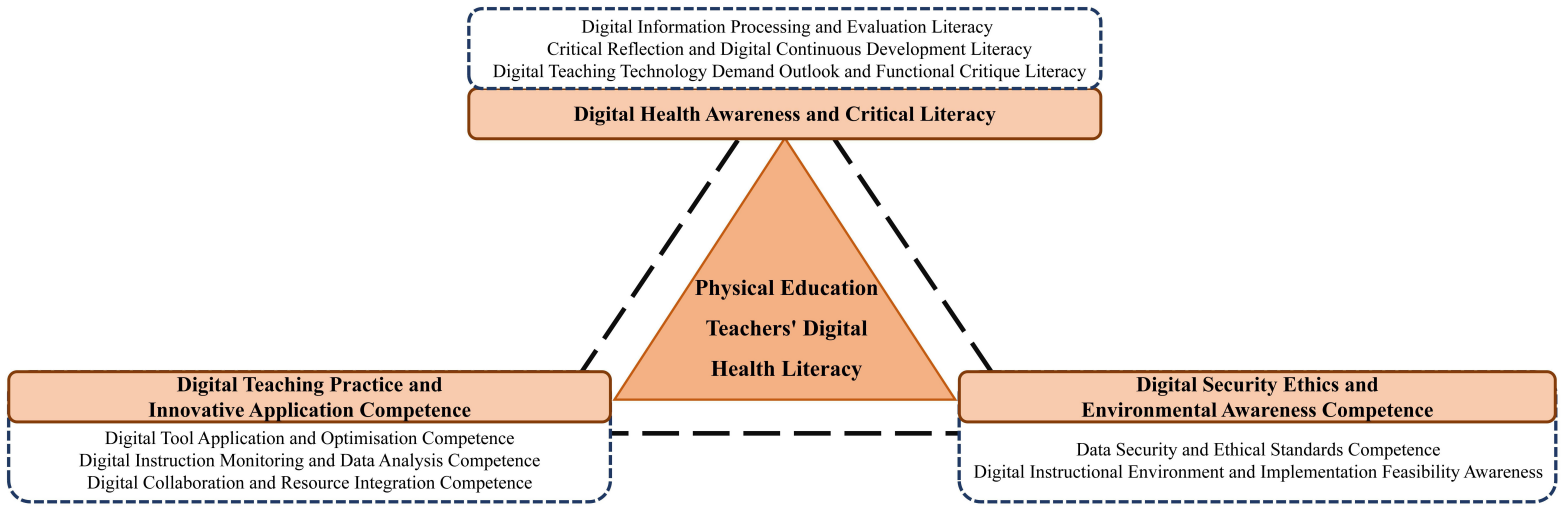
Digital health literacy model for primary and secondary school physical education teachers.

The Digital Health Literacy Framework for Primary and Secondary School Physical Education Teachers comprises three dimensions, eight categories, 40 concepts and 214 statements (Figure 1):

(a) Digital Health Awareness and Critical Literacy, including digital information processing and evaluation literacy, critical reflection and continuous development literacy and digital teaching technology requirements outlook and functional critique literacy;

(b) Digital Teaching Practice and Innovative Application Competence encompass digital tool application and optimisation in teaching, instructional monitoring and data analysis as well as digital collaboration and resource integration;

(c) Digital Security Ethics and Environmental Awareness Competence encompass awareness of data security and ethical standards competence alongside understanding of the digital instructional environment and implementation feasibility awareness.

This structural framework illustrates that digital health literacy for primary and secondary school PE teachers represents a comprehensive professional competency system. It integrates three core dimensions, namely, cognitive critique, practical application and ethical environment, which collectively encompass eight essential competency domains and are operationalised through 40 specific capability indicators. The framework reflects the expanded professional requirements that are posed by the digital era and provides a systematic theoretical basis for the cultivation and assessment of PE teachers' digital health literacy.

### 3.1 Digital health awareness and critical literacy

The dimension Digital Health Awareness and Critical Literacy represents the meta-competence that underpins the entire digital health literacy framework for PE teachers. It serves as the cognitive foundation through which teachers interpret, evaluate and respond to digital health information prior to any technical or pedagogical application (46). This competence encompasses the ability to understand systematically, assess prudently and reflect critically on the value, credibility and limitations of digital health information and technologies. It enables teachers to move beyond passive information consumption toward active judgement that filters misinformation, bias and commercial health narratives (47).

Aligned with UNESCO's conceptualization of digital literacy, which positions critical thinking as a core component, this dimension highlights that digital competence extends beyond technical skills to include the ability to question, interpret and evaluate digital content (48). Accordingly, this competence positions teachers as gatekeepers of digital health information by equipping them to discern authenticity and assess the educational potential and risks that are associated with emerging technologies.

#### 3.1.1 Digital information processing and evaluation literacy

The category Digital Information Processing and Evaluation Literacy constitutes the foundational skillset that enables PE teachers to navigate and utilize digital health resources effectively (49). It refers to the ability to locate information systematically across diverse channels, such as academic databases, professional platforms and online courses, and assess its reliability, scientific validity and timeliness. This competence allows teachers to filter and translate complex digital content into accessible knowledge for school-based health education (50). Given the prevalence of information overload, teachers must not only recognize authoritative sources but also distinguish pseudoscientific and commercially driven content. Without such discernment, educators risk inadvertently transmitting inaccurate information to students, thereby undermining their long-term health judgement (51). Therefore, this competence requires ongoing engagement with current developments in the field and the use of various strategies, such as cross-verifying, assessing source credibility and comparing claims against established scientific principles.

One teacher outlined their screening criteria: *When filtering digital health information, I adhere to standards of authority, timeliness and professionalism, prioritizing research from government official releases, international authoritative organizations and university research institutions; ensuring information is published within the last three years* (P12-2). Another educator shared their verification practice: *Information encountered can be cross-verified. For instance, upon seeing data from a fitness app, I compare it against scientific standards in textbooks. Where uncertainty persists, I consult colleagues within the teaching research group for their perspectiv*es (P2-4).

#### 3.1.2 Critical reflection and digital sustainability literacy

The category Critical Reflection and Digital Sustainability Literacy refers to teachers' capacity for the ongoing, systematic evaluation of their digital teaching practices, including assessing outcomes, identifying limitations and optimizing processes. This competence supports teachers' long-term professional development by enabling them to adapt to evolving technological trends and avoid digital practices becoming outdated, fragmented or misaligned with learner needs (52).

It also encompasses teachers' awareness of the need to update their digital health literacy continuously through professional training, academic exchange or self-directed learning. Teachers maintain a knowledge base that remains current and pedagogically relevant by actively monitoring emerging technologies and health trends. In this sense, teachers are positioned not merely as technology users but as reflective practitioners who iteratively refine their digital practices to sustain their professional competence in dynamic digital environments.

*If a tool fails to yield results, one may adjust teaching methods by combining traditional and digital approaches* (P3-12). Another educator emphasized the imperative of continuous learning: *As physical education teachers, we must keep pace with the times. AI evolves so rapidly that those who fail to keep abreast will inevitably be left behind* (P2-11). Regarding development pathways, one teacher suggested: *I believe we should first increase our exposure to these tools in daily practice, then participate in offline training to enhance collective awareness of digital health. Subsequently, we can pursue learning through diverse channels* (P1-36).

#### 3.1.3 Digital teaching technology requirements outlook and functional critique literacy

The category Digital Teaching Technology Requirements Outlook and Functional Critique Literacy refers to educators' ability to anticipate, articulate and critically evaluate the functional requirements of digital health tools in teaching contexts. This competence marks a shift from passive technology use toward actively shaping technological development to align with pedagogical principles and learner needs (53).

It involves specifying clear instructional requirements, critically assessing tool design, including privacy protection, algorithmic transparency and interface usability, and recognizing how embedded system biases may influence learner data, behavioral nudging or access inequalities (54). Grounded in classroom practice, educators are positioned to formulate expectations that extend beyond existing market offerings, thus contributing practitioner-informed critiques and recommendations. This outcome reflects a transition from technology adopters to collaborative contributors in digital tool development, thereby enhancing relevance and usability through pedagogical insight (55).

*It is hoped that future fitness trackers will not only monitor heart rate but also integrate students' physical condition data to provide personalized rest recommendations* (based on summaries from P18-15). Concurrently, teachers demonstrated strong critical awareness particularly regarding privacy concerns: *All educational apps must explicitly state how data is utilized*. (This is nonnegotiable based on summaries from P9-19, P5-31, etc.). Some educators questioned the practical utility of such tools: *Advanced digital health technologies like motion capture are better suited for professional team training... For most ordinary students, the focus should remain on physical fitness enhancement* (P27-8).

### 3.2 Digital teaching practice and innovative application competence

The dimension Digital Teaching Practice and Innovative Application Competence represents the operational core of digital health literacy, where digital knowledge is translated into concrete pedagogical action within school settings (56). It denotes teachers' ability to apply digital health tools, data and resources to design personalized instruction, integrate interdisciplinary activities and foster interactive and data-informed learning experiences. When effectively enacted, this competence enhances students' health literacy and their agency in managing health-related behaviors (57).

Aligned with the perspectives of the Organization for Economic Co-operation and Development (OECD), digital competence is ultimately demonstrated not through tool usage alone but through the generation of innovative teaching practices and improved student learning outcomes (58). Therefore, this dimension underscores the transformation of digital capacity into pedagogically meaningful and outcome-oriented instructional practice.

#### 3.2.1 Digital tool application and optimisation competence

The category Digital Tool Application and Optimisation Competence constitutes the foundational operational layer of digital teaching practice, which enables the translation of digital health concepts into concrete instructional actions and measurable learning outcomes (59). It refers to teachers' ability to select, evaluate and apply educational technology tools systematically in alignment with teaching goals and learner characteristics. This competence allows teachers to adapt digital tools to diverse learner profiles, such as differences in ability, motivation or learning styles, thereby enhancing instructional responsiveness and effectiveness (60). It further includes designing tailored digital health programmes based on students' physical fitness, health status and interests to optimize teaching outcomes.

*When evaluating new digital health tools, we first conduct basic trials to test compatibility with school teaching equipment... followed by small-scale pilot implementations... observing student feedback, data accuracy and the extent to which it enhances teaching outcomes* (P12-4). Personalized applications demonstrate rich diversity: *We set ‘stepwise heart rate targets' (gradually increasing from 120 to 140 beats per minute) via smart wristbands for students with low fitness levels* (P4-28); *Digital tools detect health data, identify issues and guide improvements; teaching adapts to individual needs based on physical assessment results* (P4-11).

#### 3.2.2 Digital instruction monitoring and data analysis competence

The category Digital Instruction Monitoring and Data Analysis Competence is essential for implementing precise and data-driven health education. It enables teachers to move from intuition-based to evidence-informed instructional decision-making, thus ensuring that pedagogical adjustments are grounded in measurable learning indicators (61). This competence involves using smart devices to collect real-time digital health data, such as heart rate or exercise load, during PE sessions, followed by quantitative analysis, visualization and progress tracking at individual and group levels. Such data-driven feedback improves instructional accuracy and enhances students' self-awareness by enabling them to visualize their own health performance (62–65). It thereby supports instructional decisions and strengthens the shift from experience-based to data-driven teaching.

*During long-distance running training with pupils... pupils wear heart rate monitors. While running, they understand their performance metrics and appropriate heart rate levels, enabling them to adjust their pace based on real-time data* (P2-12). Another teacher highlighted the role of data analysis in assessing student progress: *Examining specific metrics, such as improvements in 1,000-meter times, alongside fatigue recovery data (e.g., post-exercise heart rate changes); one pupil's 1,000-meter time didn't improve, but their heart rate recovered more quickly, indicating enhanced cardiorespiratory fitness* (P4-19).

#### 3.2.3 Digital collaboration and resource integration competence

The category Digital Collaboration and Resource Integration Competence serves as a vital means to expand the boundaries of health education and enhance teaching effectiveness. Through collaborative platforms, educators can codevelop digital health materials, exchange pedagogical strategies and collectively respond to emerging student health needs (66–68). It encompasses the ability to integrate and apply interdisciplinary teaching resources, actively participate in digital collaboration and knowledge sharing within professional communities and leverage home-school partnerships to implement digital collaborative practices. This competence embodies principles of systems thinking and open collaboration.

The ability to integrate and apply teaching resources across disciplines represents a significant and new pedagogical approach in PE. *Physical education itself is inherently interdisciplinary, encompassing fields such as exercise physiology and psychology. When analyzing sports injury data with the school doctor, we identified a general lack of flexibility in one class and promptly incorporated stretching exercises into the curriculum* (P4-22). Professional collaboration takes diverse forms: *We exchange digital health experiences with peers and initiate ‘digital health workshops' within teaching groups to share insights on digital tool usage* (P14-5). Home-school collaboration presents opportunities and challenges: *Physical education teachers communicate infrequently with parents; our current strategy involves sharing vetted educational videos in parent groups... but we avoid recommending specific apps to prevent commercial disputes* (P4-23).

### 3.3 Digital security ethics and environmental awareness competence

The dimension Digital Security Ethics and Environmental Awareness Competence provides the ethical and regulatory foundation for digital health literacy, thereby ensuring that technological applications in education remain compliant, secure and sustainable. This competence is critical for preventing data misuse, algorithmic bias and unintended psychological or behavioral risks within school environments (2, 69–71). Grounded in the ethical principles of the EU's General Data Protection Regulation (GDPR), it emphasizes safeguarding student data, prioritizing wellbeing and establishing clear accountability mechanisms to guide educators' and institutions' digital practices (72, 73).

#### 3.3.1 Data security and ethical standards competence

This competence constitutes the ethical baseline for digital health practice, thus reflecting teachers' understanding of and ability to implement data management protocols, security safeguards and privacy requirements in educational contexts. It ensures that the integration of digital technologies does not infringe upon fundamental rights, particularly students' autonomy over their personal data and digital identities (74–76). Teachers demonstrate knowledge of key principles, such as data minimization, anonymisation and encrypted storage, and are expected to act as responsible data stewards. Student health data are leveraged to enhance educational value while upholding stringent ethical standards.

*Ensuring student data security involves collecting only data directly relevant to teaching, avoiding sensitive information such as names and home addresses and using anonymous identifiers when interpreting fitness tracker data* (P14-7). Another educator proposed specific measures: *Adhering to the principle of minimal data collection, gathering only health data essential for teaching, employing end-to-end encrypted tools for data storage and transmission and signing stringent data confidentiality agreements with third-party service providers* (P11-7). Data misuse risks are also clearly recognized: *The proliferation of digital health technologies may lead to the misuse of health data, such as commercial entities leveraging health monitoring to collect students' behavioral patterns and physical data for targeted advertising or precision marketing* (P12-8).

#### 3.3.2 Digital instructional environment and implementation feasibility awareness

This competence reflects teachers' practical awareness of the contextual conditions that are required for meaningful technology adoption; it emphasizes that the integration of digital tools in PE must align with students' developmental readiness, curriculum requirements and pedagogical goals (77–79). Educators recognize that digital tool implementation is constrained by multiple objective factors and cannot occur uniformly across settings. They demonstrate sensitivity to identifying barriers, such as limited hardware infrastructure, budget restrictions, unstable network conditions and regional disparities; therefore, these factors underscore that effective technology integration depends on selecting tools that match the actual resources and circumstances of schools (80–82).

*The fundamental contradiction is: parents prioritize their children's basic needs over data privacy awareness... Shared devices among pupils are commonplace... Current baseline: resolutely avoid tools requiring facial recognition* (P4-32). Another teacher highlighted economic and hardware barriers: *The adoption of digital health technologies is constrained by school budgets; some well-funded institutions can equip themselves with smart devices... while underfunded schools can only utilize them during open lessons* (P33-7). Additionally, d*isparities in teacher skills were identified as a factor: ‘Senior teachers learn at a slower pace'* (P10-13) and ‘*there is a need to enhance skills in operating digital tools'* (D34).

## 4 Discussion

### 4.1 Intrinsic logical connections amongst literacy dimensions: an interdependent organic system

The framework developed in this study demonstrates that digital health literacy among primary and secondary school PE teachers constitutes an integrated system rather than a simple aggregation of discrete dimensions. Compared with broader international digital literacy frameworks, such as the European Digital Competence Framework (DigComp), our model shifts from “general technology use” to “pedagogical-health integration.” While international models often treat health literacy as a subset of digital citizenship, this framework positions it as a core professional competency for physical educators. The three primary categories collectively form a coherent cognition–practice–assurance cycle.

Digital Health Awareness and Critical Literacy (B1) provides the cognitive foundation, which shapes teachers' judgement, interpretive depth and decision-making in the selection and use of digital health technologies.

Digital Teaching Practice and Innovative Application Competence (B2) represents the operational core, which involves translating cognitive insights into concrete pedagogical actions and generating measurable educational value.

Digital Security Ethics and Environmental Awareness Competence (B3) constitutes the regulatory and protective layer, which ensures that digital practices remain safe, ethical, contextually feasible and aligned with institutional constraints.

These three dimensions are interdependent and mutually reinforcing. Without critical cognition (B1), digital practice (B2) risks becoming superficial or misaligned. Without practical competence (B2), cognitive understanding (B1) cannot be actualised. Without ethical and contextual safeguards (B3), innovative practices (B2) may become unsafe, inequitable or unsustainable.

Accordingly, future professional development for PE teachers should adopt a systematic approach that strengthens the coordinated development of all three dimensions, thus ensuring their integration into a comprehensive and sustainable digital health literacy framework (80–82).

### 4.2 Responding to the digital health education transformation: redefining physical education teachers' professionalism

The construction of this framework is deeply embedded in China's unique cultural and policy context. Under the “Healthy China 2030” national strategy and the “Double Reduction” policy, PE teachers are assigned unprecedented responsibility for student health management. Amidst the global shift toward educational digitalisation and the recognition of health literacy as a core 21st-century competency, the framework developed in this study directly responds to emerging expectations (83). This perspective aligns with the broader movement toward integrated open and digital education systems. In the Chinese context, where schools serve as the primary site for health intervention, the integration of digital health literacy is not merely a technical upgrade but a response to the institutional demand for data-informed health promotion. It demonstrates that digital-era professionalism requires the integration of technological competence with health-oriented pedagogical judgement (84, 85).

Traditionally, PE teacher professionalism has emphasized athletic expertise and instructional techniques. However, this framework shows that in the digital age, professionalism is increasingly enacted through new roles, including critical evaluators of digital health information, data-informed instructional decision-makers and guides for ethical and responsible digital health behavior. This development aligns with global policy directions, including the WHO's call to embed health literacy across all policy domains and the OECD's emphasis on preparing teachers as design agents who are capable of leading educational innovation (86–88). These trends collectively highlight that teachers are now positioned as mediators of digital transformation and health promotion. Specifically, the Chinese context necessitates that PE teachers navigate a digital ecosystem characterized by high mobile penetration and centralized health data platforms, requiring a unique blend of technical agility and ethical responsibility.

### 4.3 Theoretical framework and application prospects: empowering professional development and driving international dialogue

The value of this framework lies in its dual function as an explanatory theoretical model and a practical tool for guiding capacity development. To operationalize this framework in teacher training programs, a “three-tiered” approach is recommended. For pre-service teachers, training should focus on Dimension B1 (Awareness) by embedding digital health appraisal into university curricula. For early-career teachers, the focus shifts to Dimension B2 (Practice) through simulated data-driven PE teaching scenarios. Finally, for senior teachers and administrators, Dimension B3 (Ethics and Environment) should be integrated into leadership and policy-making certification.

At an operational level, it provides education administrators, school leaders and teacher training institutions with a clear roadmap for strengthening teachers' digital health literacy. For Digital Health Awareness and Critical Literacy (B1), professional learning may focus on tracing digital health information sources, evaluating evidence quality and understanding data ethics. For Digital Teaching Practice and Innovative Application Competence (B2), project-based training can support teachers in designing interdisciplinary health units that integrate wearable technologies and data-informed instruction. By providing these specific pathways, the framework moves from a static model to a dynamic tool for institutional change.

At an academic level, the framework offers a shared conceptual foundation and potential basis for developing standardized measurement tools. It enables cross-national comparative research on PE teachers' digital health literacy, supports policy benchmarking and facilitates knowledge exchange across cultural contexts. Consequently, the framework contributes not only to enhancing professional development systems but also to promoting international dialogue on digital health education within the global PE community.

### 4.4 Research limitations and future directions

This study has several limitations. Although grounded theory procedures were implemented rigorously, the broad scope of the research required a diverse sample. However, the resulting sample size may be limited. Given that the qualitative data were drawn from specific national and regional contexts, the universality and contextual variations of the proposed model require further validation across broad cultural and policy environments. Specifically, cross-national comparative studies would be invaluable to help validate the internal consistency of the identified dimensions and refine the framework's categories for broader application across diverse global educational systems.

Additionally, the framework developed for this research primarily presents a structural model of literacy. Future research should extend this work by constructing standardized assessment instruments for large-scale quantitative validation and examining how the framework's dimensions interact to influence students' health literacy and health behaviors. Based on the 40 concepts identified in this study, the research team has initiated a second phase to develop a standardized assessment scale following psychometric protocols.

Future studies may pursue several directions:

(1) longitudinal research to trace the developmental trajectory of this literacy framework across different stages of teachers' careers; Based on the 40 concepts identified in this study, the research team has initiated a second phase to develop a standardized assessment scale following psychometric protocols

(2) design-based research to develop and evaluate professional development interventions grounded in the model;

(3) cross-national comparative studies to explore the similarities, differences and underlying mechanisms of digital health literacy development among PE teachers across diverse educational systems and cultural contexts, thereby validating the framework's cross-cultural utility and refining its indicators for wider international relevance.

## 5 Implications for policy and practice

The value of this framework lies in its potential to inform systemic policy-making beyond individual classroom practice. To strengthen digital health literacy at the national level, education authorities should first prioritize the integration of these 40 capability indicators into the official “PE Teacher Competency Standards.” This would provide a formal regulatory basis for evaluating and certifying digital-era physical educators. Furthermore, the framework provides a modular structure for national-level professional development initiatives, such as the “National Teacher Training Program” in China. At an operational level, it provides education administrators, school leaders and teacher training institutions with a clear roadmap. Policies should mandate a shift from generic ICT training to the “cognition–practice–assurance” cycle identified here, ensuring that PE teachers can critically filter health misinformation (B1) and design data-informed pedagogical rituals (B2) while acting as responsible data stewards (B3). Finally, the “three-tiered” approach recommended in this study offers a practical implementation strategy for policy-makers. By embedding digital health appraisal into pre-service university curricula and integrating data-driven teaching into in-service rank promotion requirements, the framework can be operationalized as a dynamic tool for sustainable institutional change.

## 6 Conclusion

This study constructed an empirically derived digital health literacy framework for PE teachers. The model reveals that this literacy is an interconnected system encompassing critical awareness, data-informed practice, and ethical judgement. These dimensions allow teachers to navigate, apply, and evaluate digital technologies effectively.

Beyond theory, the framework serves as a catalyst for professional development. It underscores the need for coordinated capacity building. As digital health evolves, this framework provides a strategic roadmap for supporting teachers' roles and promoting student wellbeing.

## Data Availability

The original contributions presented in the study are included in the article/Supplementary material, further inquiries can be directed to the corresponding author.
