# Supplementary material for: Digital health literacy framework for PE teachers: a grounded theory study
Source: Front Public Health. 2026 Apr 28;14:1771020. doi: 10.3389/fpubh.2026.1771020 (PMC13161172; doi:10.3389/fpubh.2026.1771020)
Supplement: Supplementary file 1 [file Data_Sheet_1.pdf]

Supplementary Material

Digital Health Literacy Framework for PE Teachers A Grounded Theory Study

Yu Yang, Weijin Shi*

*** Correspondence:** Weijin Shi   shirun25@shnu.edu.cn

1 Interview Outline

Interview Outline for Exploring the Structure of Physical Education Teachers' Digital Health Literacy

Dear Teacher,

Greetings! With the deepening implementation of the "Healthy China 2030" Plan Outline, health literacy, as a strategic cornerstone for building the national public health service system, is increasingly gaining widespread attention from academia and various sectors for its theoretical value and practical significance. Against the backdrop of the deepening advancement of the technological revolution and industrial transformation, digital health literacy has become a crucial component of the modern health literacy system. This competency requires individuals to effectively achieve the precise acquisition, in-depth understanding, scientific evaluation, and effective dissemination of health information and technologies within increasingly complex digital environments, and to creatively apply them for health maintenance and promotion throughout the life cycle.

As key practitioners in health education, the level of digital health literacy among physical education teachers directly influences the effectiveness of health knowledge dissemination and pedagogical innovation. Especially within the process of digital transformation in the education system in the new era, enhancing this literacy is of significant importance for implementing the "Healthy China" strategy.

Given your extensive experience and profound insights into teacher development within the field of physical education, we sincerely invite you to participate in this research interview. Your frontline teaching experience and professional perspectives will provide crucial empirical evidence for research on physical education teachers' digital health literacy. This interview is conducted completely anonymously. The data collected will be used solely for academic research, adhering strictly to ethical principles of confidentiality. Please feel assured in providing your responses!

Appendix: *List of Questions*

1.Through which channels do you typically obtain information or resources related to digital health? (e.g., academic journals, online courses, seminars, professional books, etc.)

2.When acquiring digital health knowledge and tools, how do you screen and judge the reliability of the information?

3.How do you understand the core concepts of digital health literacy? How are these concepts reflected in your teaching?

4.When you encounter new digital health tools or technologies, what methods do you typically use to evaluate their effectiveness and applicability?

5.In your daily teaching work, do you communicate and share experiences and views regarding digital health with others?

6.In daily teaching, do you utilize digital health tools or technologies to guide students? Could you provide a practical example of an application?

7.In the process of using digital health technologies, how do you ensure the security of student data?

8.In your view, could the widespread adoption of digital health technologies potentially lead to the misuse of health data? If so, what is your perspective on this potential risk?

2 Research Informed Consent Form

Greeting！

I am currently undertaking a research project entitled “ *Digital Health Literacy Framework for PE Teachers A Grounded Theory Study”* . I would be most grateful if you would consider participating in this study by sharing your insights and suggestions, which will be invaluable to its development. You retain complete autonomy over whether to participate. We shall fully respect your decision. Please find below relevant information for your consideration:

1. Research Origins and Funding Support

This study originates from a research project supported by the Ministry of Education's Humanities and Social Sciences Research Programme (Project Approval Number: 18YJC890033).

2. The research significance is as follows:

(1) Theoretical Significance:

a. Research on physical education teachers' digital health literacy expands the theoretical framework for health education and teacher professional development.

b. It enriches studies on digital health literacy within educational contexts, providing structured theoretical support for integrating digital health into school health education.

(2) Practical Significance:

a. It provides a structured framework for assessing and enhancing physical education teachers' digital health literacy, supporting their professional development and teaching innovation.

b. It offers evidence-based reference points for designing teacher training programmes, improving teaching quality, and formulating digital health education policies.

3. We believe that your comments and suggestions will provide valuable ideas for this paper.

4.The information you fill in will only be used for the purpose of this paper, and weguarantee that we will not disclose your personal information.

Thank you for your full support and cooperation, If you have any questions,please feel free to contact us.
